# No Detectable Electroencephalographic Activity After Clinical Declaration of Death Among Tibetan Buddhist Meditators in Apparent Tukdam, a Putative Postmortem Meditation State

**DOI:** 10.3389/fpsyg.2020.599190

**Published:** 2021-01-28

**Authors:** Dylan T. Lott, Tenzin Yeshi, N. Norchung, Sonam Dolma, Nyima Tsering, Ngawang Jinpa, Tenzin Woser, Kunsang Dorjee, Tenzin Desel, Dan Fitch, Anna J. Finley, Robin Goldman, Ana Maria Ortiz Bernal, Rachele Ragazzi, Karthik Aroor, John Koger, Andy Francis, David M. Perlman, Joseph Wielgosz, David R. W. Bachhuber, Tsewang Tamdin, Tsetan Dorji Sadutshang, John D. Dunne, Antoine Lutz, Richard J. Davidson

**Affiliations:** ^1^Center for Health Minds, University of Wisconsin-Madison, Madison, WI, United States; ^2^Men-Tsee-Khang/TMAI, Upmuhal, Dharamshala, Himachal Pradesh, India; ^3^School of Human Ecology, University of Wisconsin-Madison, Madison, WI, United States; ^4^Delek Hospital, Gangchen Kyishong, Dharamshala, Himachal Pradesh, India; ^5^Department of East Asian Languages and Literature, University of Wisconsin-Madison, Madison, WI, United States; ^6^Lyon Neuroscience Research Centre, INSERM U1028, CNRS UMR5292, Lyon 1 University, Lyon, France; ^7^Departments of Psychology and Psychiatry, University of Wisconsin-Madison, Madison, WI, United States

**Keywords:** brain death, postmortem, EEG, mismatch negativity, auditory brainstem response, tibetan buddhism, meditation, consciousness

## Abstract

Recent EEG studies on the early postmortem interval that suggest the persistence of electrophysiological coherence and connectivity in the brain of animals and humans reinforce the need for further investigation of the relationship between the brain’s activity and the dying process. Neuroscience is now in a position to empirically evaluate the extended process of dying and, more specifically, to investigate the possibility of brain activity following the cessation of cardiac and respiratory function. Under the direction of the Center for Healthy Minds at the University of Wisconsin-Madison, research was conducted in India on a postmortem meditative state cultivated by some Tibetan Buddhist practitioners in which decomposition is putatively delayed. For all healthy baseline (HB) and postmortem (PM) subjects presented here, we collected resting state electroencephalographic data, mismatch negativity (MMN), and auditory brainstem response (ABR). In this study, we present HB data to demonstrate the feasibility of a sparse electrode EEG configuration to capture well-defined ERP waveforms from living subjects under very challenging field conditions. While living subjects displayed well-defined MMN and ABR responses, no recognizable EEG waveforms were discernable in any of the *tukdam* cases.

## Introduction

Over the past several decades, a combination of groundbreaking technological advances and increasingly sophisticated analytic strategies have made it possible to deploy more nuanced assessments of minimally conscious states in deeply comatose and peri-mortem patients (Wijnen et al., 2007; Kroeger et al., 2013; Di Perri et al., 2014; Gosseries et al., 2014). These same advances raise questions about conventional notions of personal identity, compound ethical issues surrounding artificial life support and organ donation, and increasingly complicate our understanding of the boundary between life and death (Lock, 1996, 2002a; Adams et al., 2009). Medical science has responded to this complex landscape by parsing brain death into distinct types, e.g., whole brain death, brainstem death (Shewmon, 2001; Lock, 2002a). Yet, such distinctions have become increasingly difficult to maintain in the wake of advances which make it possible to artificially prolong the body’s basic functions in ever more complex circumstances (Laureys, 2005; Staff and Nash, 2017; Koenig and Kaplan, 2019). Some researchers have suggested that equating “brain death” with death itself is symptomatic of a wider cultural discourse that takes a reductive, brain-centric approach to human life, personhood, and somatic integrity (Shewmon, 2001; Vidal, 2009). These tensions are further exacerbated by the ways in which declarations of death are interwoven with the demand for organs for transplant (Lock, 2002b, 2016; Gardiner and Sparrow, 2010), a profit driven health care system, and systemic problems of access, particularly for people of color (Aviv, 2018) and communities already weakened by economic devastation; realities brought into stark relief by the current pandemic. Therefore, close and socio-culturally informed examination of neural peri-mortem processes holds increasing scientific, cultural, and ethical importance.

From the perspective of the neuroscience of consciousness, recent EEG studies on the early postmortem interval reinforce the need for further investigation of the relationship between the brain’s activity and the dying process. Studies suggest the persistence of electrophysiological coherence and connectivity in the brains of rats for a period of time following induced fatal cardiac arrest (Borjigin et al., 2013), and the presence of high amplitude slow wave EEG signals for up to 80 seconds following their decapitation (van Rijn et al., 2011). Studies in humans have also shown evidence of surges in EEG and the bispectral index (BIS) at or in the period following clinical death (Chawla et al., 2009; Auyong et al., 2010; Norton et al., 2017). Recently, research using the mismatch negativity (MMN) paradigm suggests that the auditory pathways may be responsive to stimuli in the moments just prior to clinical death (Blundon et al., 2020). Altogether, these studies strongly suggest that death is not an event that occurs at a single point in time.

While scientific research has primarily studied neural processes in the immediate window surrounding clinical death, many of the world’s religions and cultures have long held that death is a process extended in time (Tylor, 1958; Palgi and Abramovitch, 1984; Boyer, 2007; Gennep, 2010). Neuroscience is now in a position to empirically evaluate the extended process of dying and more specifically, to investigate the possibility of brain activity following the cessation of cardiac and respiratory function. Whether this would also imply that there is some form of subjective experience that persists beyond the cessation of cardiorespiratory function is, at this time, merely a matter of speculation. However, the possibility that brain activity—along with the concomitant possibility of some level of consciousness—persists in the period immediately following clinical death raises a host of questions: are there factors or behaviors that effect how long postmortem brain activity persists? How might we objectively assess or demonstrate the presence of subjective consciousness during that time or parse its chronometry? If it becomes possible to demonstrate the persistence of some form of brain activity that has previously been correlated with subjective awareness, what form of ethics should guide how we relate to the decedent body?

The Center for Healthy Minds at the University of Wisconsin-Madison was invited to explore some of these questions in India through research on a postmortem meditative state cultivated by some Tibetan Buddhist practitioners. Tibetan Buddhists believe that this state, known as *tukdam* (*thugs dam*), enables one to achieve spiritual liberation by experiencing the fundamental nature of mind that is said to be especially accessible at the time of death, when the mind is believed to no longer register sensory impressions or engage in conceptual elaboration. All humans are said to have this opportunity, as it is believed to arise naturally during the process of dying, but only advanced meditators are thought to have the ability to apprehend and use that experience for spiritual realization (Lati Rinpoche et al., 1979; Dalai Lama XIV and Hopkins, 2004; Chagme and Rinpoche, 2010; Tsenshap et al., 2011). According to the tradition, this meditative state mainfests externally as a delay in, or attenuation of, the processes of postmortem decomposition. The visage of those in *tukdam* is described as *radiant*, their skin remains supple and elastic, and the area around the heart is said to be warmer than the rest of the body. Individuals in *tukdam* “… can remain in this state for a week or even a month according to their own wish … Even in the hot season in India people have remained in [this meditative state] for two weeks like someone asleep – no longer breathing, like a corpse but, unlike a corpse, not smelling.” (Dalai Lama XIV, 2006:177-178). When the body starts to smell and signs of bodily decomposition become apparent, it is understood that *tukdam* has been released.

This *Tukdam* Project, developed in conversations between Dr. Richard J. Davidson and His Holiness Tenzin Gyatso, the XIV Dalai Lama, is a collaborative long-term empirical research effort of the Center for Healthy Minds in partnership with Men-Tsee-Khang (Sowa-Rigpa, Dharamsala, India), Delek Hospital (Dharamsala, India), and the Office of His Holiness the Dalai Lama. Informed by Tibetan Buddhist medical and religious understandings of the death process, the project combines ethnographic and psychophysiological research in an attempt to understand the social and meditative practices that Tibetan Buddhists believe provide the foundation for entering *tukdam*. It also seeks to discover what neural or other biological mechanisms may be involved in postmortem cases recognized by Tibetan Buddhists as *tukdam*. We hypothesized that some residual brain activity might persist in *tukdam* practitioners for a period following clinical death and that this might be a factor associated with the delay in decomposition which—although culturally recognized—has not yet been confirmed through objective measures. To detect such activity, we used EEG to monitor the brain’s response to auditory stimuli. This approach is supported by studies that show specific types of auditory stimuli elicit well-defined electrical signals (event related potentials) independent of conscious attention and in states of minimal consciousness (Näätänen et al., 2007). For example, MMN waveforms are discernable in patients in a vegetative state and suggest the presence of some residual cognitive activity (Boly et al., 2011). Additionally, the auditory brainstem response (ABR) has been used to determine the viability of the brainstem in cases of coma and brain death (Hall et al., 1985; Kaga et al., 1985; Garcia-Larrea et al., 1987; Facco et al., 2002). In this report, we present the initial findings of our continuing research on possible EEG correlates of *tukdam*. To demonstrate that we are able to collect quality data under challenging field conditions we also present data collected from living practitioners during the course of our research.

## Materials and Methods

### Ethics and Recruitment

Our research received approval from the University of Wisconsin’s Institutional Review Board and the Research Ethics Committee of Men-Tsee-Khang. All Men-Tsee-Khang Team members received Human Subjects Research training as well as training in all data collection and study protocols. Living subjects provided written consent after being informed of the study goals and a demonstration of the equipment and the procedures involved. Decedent subjects were enrolled in the study with the consent of the eldest family member or disciple. All participants (or their surrogates) were given the equivalent of $25 in Indian Rupees for their participation. This amount was determined as a culturally acceptable offering without incentivizing participation.

We succeeded in enrolling 14 living practitioners and 13 *tukdam* subjects. All subjects presented without a history of neurological disorder or hearing impairment beyond that associated with aging. Recruitment to the study was complicated by the fact that practitioners are very reserved and typically secretive about their practice and accomplishments, as both secrecy and humility are considered integral to sustaining the mindset upon which higher states of realization depend. Furthermore, monasteries and retreat communities in India are often separated by long distances that are challenging to traverse. To aid in recruitment, The Office of the His Holiness the Dalai Lama sent letters to 28 nunneries and monasteries representing the major Tibetan Buddhist denominations in India requesting their cooperation and help in identifying potential candidates. Team members did further outreach and demonstrations of equipment at major monasteries, religious elder homes, and community centers in Tibetan communities throughout India.

Though some healthy baseline (HB) subjects were recruited following presentations in the Tibetan monastic and lay communities, most of our practitioners were recruited through relationships and contacts developed by Dylan Lott (DL) over successive periods of fieldwork.

Between 2013 and 2018, we received notification of 13 postmortem *tukdam* cases from across India. Word of these cases were directed either to the Office of His Holiness the Dalai Lama, Dr. Tsetan Sadutshang (TS; Delek Hospital), or Dr. Tsewang Tamdin (TT; Men-Tsee-Khang). Occasionally, DL or other team members were notified first. In all cases, either TT or TS would perform an initial phone interview to determine if the subject fit our criteria (no history of neurological disease, no odor or other signs of decomposition present, the body had not been preserved in any way). If these criteria were met, TT (a Western-trained medical doctor) and TS would examine the decedent in person—or via digital images—to confirm that decomposition had not set in prior to requesting permission for our research team to observe and record. Given the distances and challenging travel conditions within India, some cases required a day’s journey before we could begin recording. Once there, we would conduct initial observations and send digital images for visual inspection by TT or TS who would make the decision whether to proceed with data collection. In some cases, the teams were welcomed to observe but ultimately not permitted to record data. In other cases, the *tukdam* had concluded and decomposition begun by the time we had reached the location.

### Experimental Design

For all healthy baseline (HB) and postmortem (PM) subjects presented here, we collected resting state electroencephalographic data, MMN, and ABR. Recording sessions in postmortem subjects were repeated twice daily, once in the morning and once in the afternoon until decomposition began or *tukdam* was deemed concluded.

For all subjects, medical records, meditation practice and life history questionnaires were collected (from family members or disciples in the case of PM). HB subjects were also queried about their lucidity and level of awareness during the time of data collection. Interviews and data collection sessions were, when feasible, video recorded by the research team.

### Recording Environments

As all data were recorded in field conditions, special note must be made regarding the recording environments, which varied greatly (see Tables 1, 2). Most *tukdam* subjects were recorded in hospital or monastery quarters, with one *tukdam* case recorded in a retreat area above Dharamsala. Living subjects were recorded in monastery quarters, retreat areas above Dharamsala, and elder homes. Table 1 presents data recorded from living subjects to demonstrate that we were able to get quality data under the same challenging conditions in which PM data was collected; it is not intended to represent a control group for the PM subjects themselves (see Discussion section). To guarantee a stable, well-conditioned power supply under local conditions, all data collection was done on battery power only. Participants were positioned—when possible and with minimal disturbance of the body—away from any conductive material or electrical outlets. We used a digital EMF meter to optimize the position of our equipment to ensure we recorded in areas that minimized electrical noise while still preserving culturally appropriate locations for the deceased. During data collection, all electrical appliances (ceiling fans, heaters, lights) were turned off and/or unplugged.

**TABLE 1:** Status of HB subjects and the location and setting in which data were recorded.

Subject	Gender	Status	Location	Setting
HB1	F	Nun	Dharamsala	Elder home
HB2	M	Monk/retreatant	Dharamsala	Room
HB3	M	Monk/retreatant	Dharamsala	Retreat area
HB4	M	Monk/retreatant	Sidhbari	Room
HB5	M	Lay Practitioner	Tilopur	Monastery

**TABLE 2:** For PM subjects: gender, status, the day following clinical death in which the research team was able to begin collecting data, the cause of death, and the setting in which data were collected.

Subject	Gender	Status	Time since clinical death	Cause of death	Location/setting
PM1	M	Monk	26 hours	Gastric cancer	Hospital
PM2	M	Monk	5 days	Aspiration pneumonia	Monastery
PM3	M	Monk	5 days	History of RCC and lung/bone metastasis	Monastery
PM4	F	Lay woman	3 days	Not specified	Residence
PM5	M	Monk	4 days	Renal failure, diabetes, Koch’s TB	Monastery
PM6	M	Monk	3 days	Gastro-intestinal bleeding	Retreat hut
PM7	M	Monk/retreatant	3 days	Lung cancer	Hospital

### EEG Recording and Processing

All EEG data were collected and recorded using Biosemi’s ActiveTwo^®^ system via a Dell Latitude E6530 (Jan 2013–May 2019) or Dell Latitude 7490 (June 2019) laptop computer. Four flat Ag/AgCl electrodes were placed on the scalp with adhesive conducting gel after cleaning each site with an alcohol pad: Fz, Cz, Pz, and T8. Biosemi’s common mode sense (CMS) and driven right leg (DRL) electrodes were used to improve impedance and reduce interference through reference to the AD-box output (van Rijn et al., 1990). These active electrodes were placed at C3 (CMS) and C4 (DRL) on the coronal plane of the scalp. Right and left mastoids were used as reference. Bioplar EKG leads were placed just beneath the midpoint of the right clavicle and below the left floating rib. All electrodes were wrapped five times by the CMS/DRL wire to provide further insulation of the signal to the AD-box.

Data were recorded at a sampling rate of 16384Hz across all phases of the protocol. Electrode impedances were monitored using Biosemi’s Actiview^®^ software. Auditory stimuli were played from a WAV file on the study laptop and were delivered using Etymotic ER-3^®^ pneumatic headphones tipped with foam inserts. The transducer housings were attached by clips to the lapel of a robe or shirt at a sufficient distance away from scalp electrodes in order to minimize possible signal interference. Auditory levels were tested for comfort and audibility with living subjects prior to running the protocol; with decedent subjects, volume was set loud enough to be heard faintly by the researcher standing two feet distant. Each auditory stimulus was registered by a custom designed digital trigger that permitted visual monitoring via Actiview^®^ of the stimuli during recording and precise epoching based on stimulus onset during analysis.

Following a 5 to 10 min baseline data collection, MMN and ABR tasks were administered. The MMN protocol was a roving odd-ball paradigm similar to Garrido et al. (2008) with a duration of 16 min and 40 s. Pure tones (70 ms) were presented every 500 ms and varied from 500-800 Hz in steps of 50 Hz, with the same tone repeating from 1 to 11 times pseudorandomly. There were a total of 2000 stimuli per session, 13% of which were deviant. One of four roving odd-ball tone series were randomly selected and presented during any given session to reduce the likelihood of attenuated response via adaptation and prediction in the event of multiple recording sessions (Garrido et al., 2009). The canonical finding with MMN is the increased negative amplitude of the negative waveform found in response to a rare deviant event presented among a set of standard stimuli. This MMN occurs irrespective of a person’s direction of attention. If the MMN was detectable postmortem, it would indicate some residual cortical activity. MMN was followed by 30 s of silence, which was in turn followed by the ABR protocol. This consisted of a series of 4000 220 “click” events (white noise, 0.1 ms duration) presented at 20 Hz (9 subjects) or jittered 17–21 Hz (3 subjects), producing 20 “clicks” per second for 3 min and 20 s. ABR reflects activity at the cochlear nucleus and in the brainstem. If the ABR was detected postmortem, it would indicate some residual brainstem activity postmortem.

### EEG Analysis

Using a study specific interface written in MNE-Python (Gramfort et al., 2013; Fitch et al., 2020), data were visually inspected for artifacts with a notch filter at 50 Hz and a highpass filter at 0.5 Hz, using the MNE filter defaults. Epochs containing pulse or eye movement, gross movement, or which were contaminated by higher frequency, higher amplitude noise were manually rejected. For analysis, data were filtered with MNE’s raw filter function defaults, using the default fir design of “firwin.” MMN data were bandpass filtered from 1 Hz to 35 Hz, epoched between −100 ms and 400 ms from stimulus onset, baseline corrected, and averaged separately for standard and deviant trials. A difference waveform was calculated by subtracting standard from deviant. The classic MMN difference wave form is negative in the range of 100 to 300 ms (Hall, 2007). Our MMN wave form was quantified as the mean amplitude of the difference waveform from 90 ms to 180 ms after stimulus onset at Fz and Cz, following previous literature in expert meditators (Fucci et al., 2018). ABR data were bandpass filtered from 100 Hz to 3000 Hz, epoched between −2 ms and 10 ms from stimulus onset, baseline corrected, and averaged across all trials. The ABR was quantified as the duration of the maximum positive peak which typically occurs as a wave IV/V complex (Hall, 2007) between 4 and 8 ms, calculated as the time between when the waveform first crossed zero before and after the peak. All MMN and ABR data were weighted by the number of trials included per subject.

### Participants

#### Healthy Baseline Subjects

Healthy baseline EEG data were collected by the same teams and in comparably challenging settings as expected for *tukdam* subjects, for 14 subjects (monastic practitioner *n* = 10, lay practitioner *n* = 1, lay non-practitioner *n* = 3). These recordings are included here to demonstrate our ability to get quality data under challenging field conditions identical to those in PM cases and are not intended as a control group. Of these, 8 were excluded either because of excessive noise, eye, or head movement and one incomplete protocol (following a request to discontinue after approximately 9 min). Additionally, one HB had a single noisy electrode (Fz), which was excluded from analyses. Practitioners were all seated and instructed to relax and not engage in any specific type of meditation; all chose to close their eyes during the recording. Of those presented in this paper, only one is female (see Discussion section). Ages range from 61 to 86 years (mean = 73.6) (Table 1).

#### Tukdam Subjects

Between 2013 and 2016, we were informed of 12 potential cases of *tukdam*, of which the research team was permitted to record five. Between 2017 and 2019, the team learned of 15 potential *tukdam* cases, of which we were permitted to record eight. Thus, PM EEG data were collected on 13 subjects in total (monastic practitioner *n* = 11, lay non-practitioner *n* = 2). Of these, six could not be included in this analysis owing to excessive electrical interference or incomplete protocols. Ages range from 43 to 91 years (mean = 70.6) (Table 2). In this report, we present data only from the recording session closest to the time of clinical death. The earliest our teams were able to record was 26 h postmortem, although 3 or 5 days were more common (see Discussion section).

## Results

### Mismatch Negativity

**FIGURE 1:**
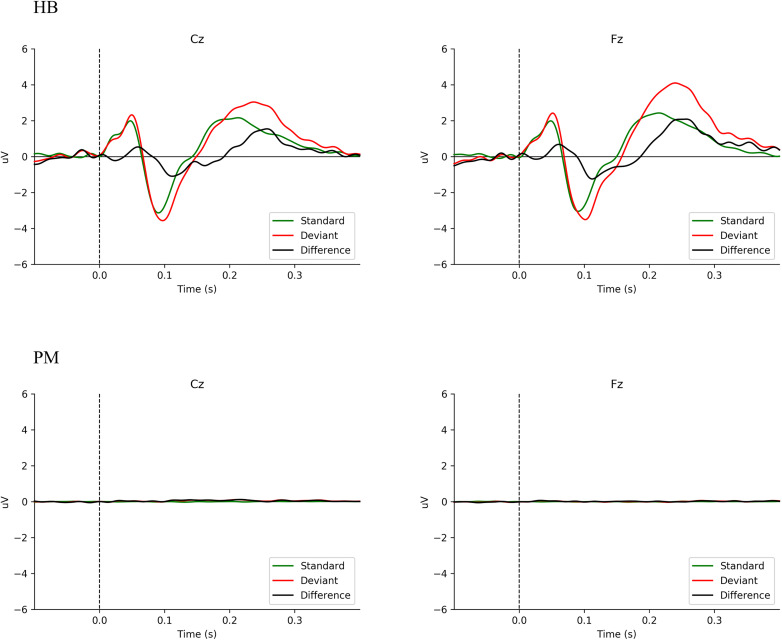
Comparison of averaged MMN wave forms for all Living Subjects (HB) and Postmortem Subjects (PM).

Figure 1 shows the averaged ERP responses for MMN (deviant minus standard = difference) in HB and PM subjects. Data are here shown for Fz and Cz, given the primary distribution area of the MMN response (Hall, 2007; Näätänen et al., 2007). In HB subjects, a typical MMN wave form is seen. Using Welch’s *t*-test on the mean amplitude of the difference waveform by group, Fz, *t*(3.07) = 3.46, *p* < 0.039; for Cz, *t*(4.27) = 5.29, *p* < 0.005. The mean difference for HB Fz = −0.71 _μ_V (sd = 0.35 _μ_V) and HB Cz = −0.71 _μ_V (sd = 0.28 _μ_V). For PM Fz = 0.01 _μ_V (sd = 0.05 _μ_V) and PM Cz = 0.07 _μ_V (sd = 0.07 _μ_V). PM subjects (Figure 1 PM) have no discernible response. In HB subjects (Figure 1 HB), a negative deflection just after 100 ms is clearly visible. Moreover, the sample size precluded any meaningful analysis by age (Pekkonen et al., 1993; Hall, 2007). Supplementary Figure 1 presents MMN data for HB and PM subjects individually.

### Auditory Brainstem Response

**FIGURE 2:**
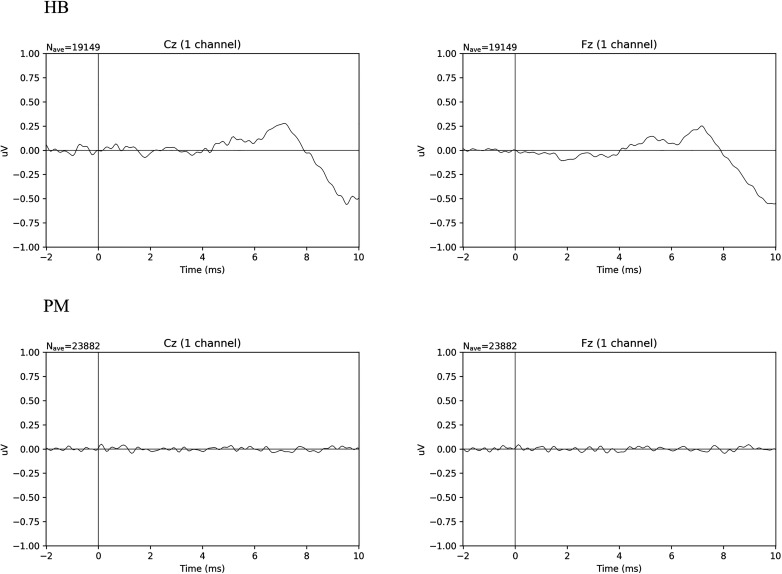
Comparison of averaged ABR wave forms for all Living Subjects (HB) and Postmortem Subjects (PM).

Figure 2 shows the averaged ERP responses for ABR in HB and PM subjects. In HB subjects, a typical ABR wave form is seen. Using Welch’s *t*-test on the duration of maximum peak of the wave IV/V complex at Fz, *t*(4.25) = 4.41, *p* < 0.010; for Cz, *t*(5.09) = 3.23, *p* < 0.022. The mean difference for HB Fz = 3.24 ms (sd = 1.25 ms) and HB Cz = 3.15 ms (sd = 1.41 ms). For PM Fz = 0.47 ms (sd = 0.27 ms) and PM Cz = 0.77 ms (sd = 0.62 ms). Data are here shown for Fz and Cz (Hall, 2007). PM subjects (Figure 2 PM) show no discernable waveform, while in HB subjects (Figure 2 HB) wave V peaks at ∼7.5 ms. The relatively low amplitude of the wave peaks in HB is consistent with models based on age-related hearing loss and the limitations of our array (Hall, 2007). Supplementary Figure 2 presents ABR data for HB and PM subjects individually.

## Discussion

In this study, we demonstrated the feasibility that a sparse electrode EEG configuration is capable of capturing well-defined ERP waveforms from living subjects under very challenging field conditions. We also presented EEG data on individuals who were recognized by Tibetan lay, medical, and religious specialists in India as being in the postmortem state called *tukdam*. No recognizable EEG waveforms were discernable in any of these *tukdam* cases, thus we failed to find support for the hypothesis of residual brain activity following the cessation of cardiorespiratory function in *tukdam* cases recorded beyond 26 h postmortem. While we did not expect to be able to assess whether some form of awareness was present (as asserted in the Tibetan Buddhist tradition), we had hypothesized that residual brainstem activity could be a factor in the reported delay of decomposition. This hypothesis was based on research demonstrating the brainstem’s role in integrating the activity of different organ systems of the body and regulating homeostasis through the management of bodily energy and wastes (Shewmon, 2001).

It is important to note that even if *tukdam* is mediated by residual electrical activity in the brainstem, this activity may generate signals that are too weak to be detected on the scalp surface or not possible to resolve owing to the limitations of our field equipment. If signal were detected, we would still need other types of data to shed light on the possible mechanisms that link brain activity and external signs of *tukdam*. Alternatively, if activity (or in this case, lack of activity) in the brain postmortem is not a mediator of the reported lack of decomposition, other biological mechanisms could be responsible. In both cases, we believe that—in addition to lifestyle, medical, and practice history—collecting blood, saliva, and tissue to investigate other potential mechanisms is key (Hyde et al., 2013; Metcalf et al., 2016). When such fluids and tissues become available, discovery-based science with large-scale metabolomics and whole epigenome arrays can be examined.

### Challenges and Limitations

There are several limitations to this study, a number of which result from the unique conditions in which the study must be carried out and from the difficulty of operationalizing the culturally salient signs of *tukdam* for research (e.g., smell, rate of decomposition, and lasting suppleness of the skin).

Although we have done extensive outreach and the Dalai Lama regularly speaks of the importance of scientific research on *tukdam* in his public talks to the Tibetan community, timely notification of *tukdam* events or of individuals nearing the time of death continues to be difficult to obtain. As noted in Table 2, the earliest we have been able to record data has been 26 h from the time of death. In part, the delay is a consequence of the typical Tibetan Buddhist practice of observing the deceased for three days before determination can be made of *tukdam.* Other factors, including the belief held by some Tibetans that touching the body too soon following death will disturb the *tukdam*, also played a role in delayed access to potential cases. In the future, by establishing relationships with practitioners when they are still alive, we hope to minimize the interval between the conventional Western definition of death and the time we begin recording. Ideally, we will seek to record practitioners—and lay persons not expected to enter *tukdam* as a control group—as they are in the process of dying and before the cardiorespiratory function has completely ceased.

Other sources of data—such as blood, saliva, and gut biome—would be important to collect as the study moves forward, but which we were unable to do given certain cultural and infrastructural challenges.

As we cannot predict when and if someone will enter *tukdam*, we also remain focused on increasing the number of HB and perimortem subjects who, based on their practice history, have a greater likelihood of entering *tukdam*. A greater number of practitioners will provide us with much needed control data and lay the foundation for the acquisition of perimortem data. Such data will enable us to examine possible predictors of entering *tukdam*, something that has eluded practitioners for centuries. Further, that we have but one female subject in each group (HB, PM) reflects the broad cultural and religious challenges we have had recruiting women to the study. Efforts to recruit female practitioners are underway.

Finally, it is important to stress that this study was not meant to adjudicate the empirical status of *tukdam per se*. As a culturally specific way of talking about a range of phenomena linked to religious narratives of liberation and a sustained philosophical speculation as to the nature of the mind and of consciousness, *tukdam* as such belongs to a very specific cultural horizon (Lévi-Strauss, 1966). Notwithstanding the complex challenges involved, taking the empirically testable claims associated with *tukdam* and rigorously investigating them offers us a unique opportunity to explore and deepen our understanding of the relationship between the brain, the body, and consciousness. Research into Tibetan Buddhist practices have already yielded a number of such insights (Benson et al., 1982; Lutz et al., 2004, 2007; Rosenkranz et al., 2019; Adluru et al., 2020). In that vein, our present research documented multiple incidences of *tukdam* and investigated with rigor the possible mechanisms involved. Although the methods used did not reveal neurophysiological correlates of the *tukdam* state, in conducting this study we identified and addressed numerous complex scientific and cultural considerations, demonstrating the feasibility of such research. This process offers a model for future investigations of meditation and the dying process across cultures.

## Data Availability Statement

The datasets presented in this article are not readily available. The protocol allows us to share data; however, each request must be reviewed by the University of Wisconsin IRB beforehand. Requests to access the datasets should be directed to RD; rjdavids@wisc.edu.

## Ethics Statement

The studies involving human participants were reviewed by and received approval from the Institutional Review Board, University of Wisconsin-Madison and the Research Ethics Committee of Men-Tsee-Khang. The participants or their surrogates provided their written informed consent to participate in this study.

## Author Contributions

DL wrote the manuscript, managed the study, conducted interviews and outreach, and collected and analyzed the data. RD edited the manuscript and conceived of the research design, methods, and analyses. AL conceived of the research and helped design methods and experiments and analyses, and helped edit the manuscript. JD helped edit the manuscript. TT and TS helped with research design and implementation and oversaw data collection in India. DB, DP, and JW contributed to the research design and methods. KA and JK helped design the field equipment, experimental procedures, and field analysis methods. DP and JW also helped edit the manuscript. AB and RR helped to process and analyze MMN and ABR data. RG, DF, and AJF designed the analysis scripts, analysis methods, figures, and statistical analyses and edited the manuscript. TY, NT, NN, SD, NJ, TW, KD, and TD collected data and provided translation and logistical support. All authors contributed to the article and approved the submitted version.

## Conflict of Interest

RD is the founder and president, and he serves on the board of directors for the non-profit organization, Healthy Minds Innovations, Inc. In addition, RD served on the board of directors for the Mind & Life Institute from 1992 to 2017. The authors declare that the research was conducted in the absence of any commercial or financial relationships that could be construed as a potential conflict of interest.
