# Supplementary figures and images for: No Detectable Electroencephalographic Activity After Clinical Declaration of Death Among Tibetan Buddhist Meditators in Apparent Tukdam, a Putative Postmortem Meditation State
Source: Front Psychol. 2021 Jan 28;11:599190. doi: 10.3389/fpsyg.2020.599190 (PMC7876463; doi:10.3389/fpsyg.2020.599190)

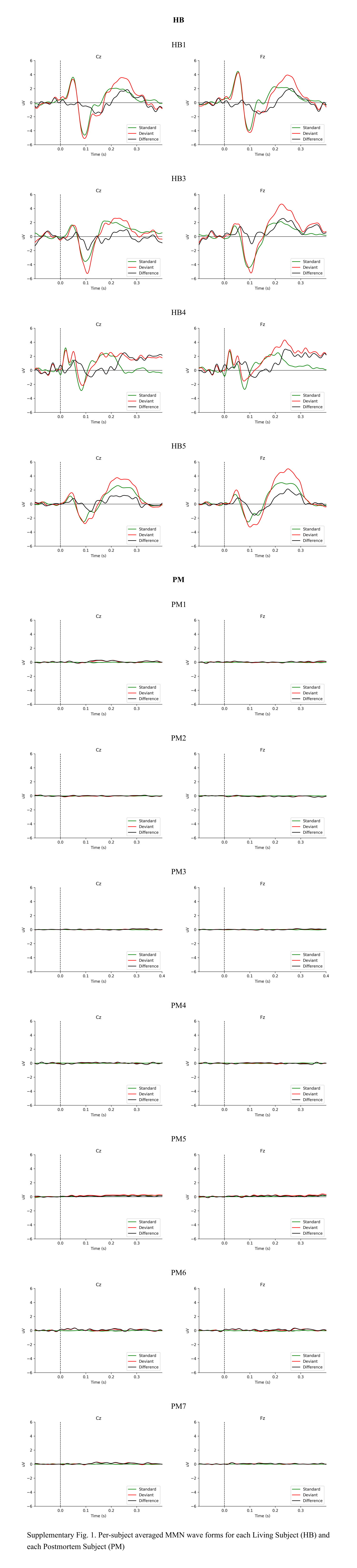

Supplement: Supplementary file 1 [file Image_1.TIF]
